# Win Ratio in Biomedical Science: A Bibliometric Analysis

**DOI:** 10.1016/j.cjco.2025.05.006

**Published:** 2025-05-21

**Authors:** Zhenyu Li, Aliya Izumi, Dominique Vervoort, Anika Ranadive, Subodh Verma, Stephen E. Fremes

**Affiliations:** a Faculty of Medicine, University of Ottawa, Ottawa, Ontario, Canada; b Schulich Heart Centre, Sunnybrook Health Sciences Centre, Toronto, Ontario, Canada; c Division of Cardiac Surgery, Department of Surgery, University of Toronto, Toronto, Ontario, Canada; d Institute of Health Policy, Management and Evaluation, University of Toronto, Toronto, Ontario; e Michael G. DeGroote School of Medicine, McMaster University, Hamilton, Ontario, Canada; f Li Ka Shing Knowledge Institute, St Michael's Hospital, Toronto, Ontario, Canada

**Keywords:** cardiology, statistics, clinical trial, win ratio, bibliometric analysis

## Abstract

**Background:**

The win ratio (WR), introduced in 2012, has emerged as a method to analyze hierarchical composite outcomes by prioritizing clinically significant events, unlike traditional composite time-to-event analyses, which treat events equally. However, use of the WR in biomedical research beyond cardiovascular trials remains unexplored. The study aims to investigate trends in the use of the WR in biomedical research and determine the characteristics of these articles.

**Methods:**

Biomedical articles indexed in Web of Science and PubMed were retrieved for 2012-2024. Data extraction included bibliometric information and content details. Statistical analyses utilized descriptive statistics, correlation, and linear regression to assess publication trends and the distribution of WR methodologies across disciplines.

**Results:**

A total of 82 studies were analyzed. Publication counts using the WR have grown significantly since its introduction, with an annual compounded growth rate of 30.2%. Most articles were randomized controlled trials (n = 68; 82.9%). Of the 68 randomized controlled trials, 46 (67.6%) were in the field of cardiology. The unmatched WR was the predominant WR approach (n = 57; 69.5%). Mortality was the highest-ranked outcome in most studies (n = 55; 67.1%), and time-to-event variables were the most frequently used across all hierarchical outcome ranks (n = 173).

**Conclusions:**

The WR has gained acceptance as a robust and clinically meaningful method for analyzing composite endpoints, particularly for cardiovascular trials. Although challenges remain, its adaptability and ability to prioritize clinically relevant outcomes make it a promising tool for future biomedical research across various disciplines.

## Résumé

**Contexte:**

Le « Win Ratio » (WR), introduit en 2012, est une méthode permettant d’analyser des paramètres composites hiérarchiques en donnant la priorité aux événements cliniquement significatifs, contrairement aux analyses composites traditionnelles basées sur le temps avant la survenue de l’événement, qui traitent tous les événements de manière égale. Son utilisation en recherche biomédicale et en dehors des études sur les maladies cardiovasculaires demeure néanmoins inexplorée. Cette étude vise à dégager des tendances dans l’utilisation du WR en recherche biomédicale et à déterminer les caractéristiques de ces articles.

**Méthodologie:**

Nous avons extrait les articles biomédicaux référencés dans Web of Science et PubMed entre 2012 et 2024. Les données extraites comprennent l’information bibliométrique et la description du contenu. Les analyses statistiques reposent sur des statistiques descriptives, une corrélation et une régression linéaire pour évaluer les tendances de publication et la distribution des méthodologies WR entre les disciplines.

**Résultats:**

Au total, 82 études ont été analysées. Le nombre de publications utilisant le WR a considérablement augmenté depuis son introduction, comme en témoigne un taux de croissance annuel composé de 30,2 %. La plupart des articles concernaient des essais contrôlés randomisés (ECR) (n = 68; 82,9 %). Parmi les 68 ECR, 46 (67,6 %) relevaient du domaine de la cardiologie. Le WR non apparié a été la méthodologie WR la plus utilisée (n = 57; 69,5 %). La mortalité a été le paramètre d’évaluation occupant le niveau le plus élevé de la hiérarchie dans la plupart des études (n = 55; 67,1 %), tandis que les variables « temps avant l’événement » ont été celles le plus souvent utilisées à tous les niveaux de la hiérarchie (n = 173).

**Conclusions:**

Le WR est aujourd’hui reconnu comme une méthode robuste et cliniquement pertinente pour l’analyse des paramètres d’évaluation composites, en particulier dans le cadre des essais sur les maladies cardiovasculaires. Bien qu’il reste des défis à relever, son adaptabilité et sa capacité à hiérarchiser les paramètres cliniquement pertinents font de lui un outil prometteur pour l’avenir de la recherche biomédicale dans diverses disciplines.

Health outcomes research focuses predominantly on hard clinical outcomes, such as mortality, hospitalization, and other complications. Outcomes are commonly assessed in isolation, and then grouped into composite endpoints for reasons of clinical relevance and statistical power and/or feasibility. Conventionally, the analysis of composite endpoints in clinical research using survival analysis emphasizes the time until the first event, giving equal importance to all outcomes included in these composites.^1^ This approach commonly employs the Kaplan–Meier estimator, the log-rank test, and Cox proportional hazards regression to evaluate event-free survival.^2^ However, this approach has limitations, such as the grouping of outcomes of varying significance (eg, death vs hospitalization), the assignment of primacy to a less important event (eg, hospitalization) when a more significant event (eg, death) occurs later, and the lack of capture of recurrent events (eg, multiple hospitalizations).^3^

To address the limitations of time-to-first-event analysis, Pocock et al. introduced the win ratio (WR) method in 2012,^1^ building on Finkelstein and Schoenfeld's pairwise comparison principle from 1999.^4^ The WR method offers a refined, flexible approach to evaluating composite endpoints by establishing a hierarchy of outcomes based on their clinical significance. The type of composite outcome can include the time to event, and recurrent events, as well as continuous and categorical outcomes.^5^ The hierarchy accounts for clinical relevance, meaning that more-severe outcomes (eg, death) are considered more significant than less-critical events, such as nonfatal endpoints, and they therefore receive priority in the hierarchy.^1^ Starting from the most critical outcome, patient-to-patient comparisons are made, proceeding down the hierarchy until a "win" is determined, depending on which patient has a more favourable clinical outcome. If no clear advantage is found after all components in the hierarchy have been assessed, the comparison is recorded as a "tie."^2^

The WR has been used commonly in cardiovascular (CV) trials.^6^ In the past, CV trials prioritized single, hard endpoints, such as all-cause mortality or CV death. However, powering trials for mortality alone requires a large sample size, which often is unfeasible to obtain.^7^ To capture a wider range of clinically relevant outcomes and for statistical convenience, CV trials increasingly began to include composite endpoints, which combine multiple outcomes (eg, myocardial infarction, stroke, hospitalization, death) into a single measure.^3^ Composite endpoints have the advantage of requiring a smaller sample size to detect statistical differences, and they are particularly useful when multiple clinical endpoints are related (eg, CV death and nonfatal myocardial infarction).^8^ In recognition of the importance of outcomes that resonate directly with patients, many CV trials also have expanded to include patient-centred outcomes, such as quality of life (QoL), symptom relief, and functional status.^9^ An advantage of the WR approach is that composite outcomes of different types of variables also can be created (eg, time-to-event, binary, ordinal, continuous, etc.). The WR method builds on the development of composite outcomes by prioritizing outcomes based on their clinical importance, allowing for a nuanced interpretation that respects the hierarchy of composite outcomes. By comparing patients in pairs and emphasizing the most clinically significant events, the WR provides a refined measure of treatment effect that aligns well with the goals of reflecting both clinical and patient-centred impacts.

The use of WR in CV trials has been described in previous studies.^2^^,^^6^ However, its use overall in biomedical literature, including in non-CV randomized controlled trials (RCTs), has not been described. Given that WR was introduced originally in cardiology and remains widely used in CV trials, understanding its broader application may provide valuable insights into its evolving role in clinical trial design and outcome prioritization. The purpose of this bibliometric review is to analyze publication trends related to WR, occurring from 2012 to 2024, and to assess its impact across biomedical disciplines while considering its continued relevance for CV research.

## Method

### Literature search

The study was registered on the Open Science Framework (OSF) (doi.org/10.17605/OSF.IO/4ME7C). A literature search was performed on PubMed and the Web of Science core collection (2012-present) during August 2024. The full search strategy is available in Supplemental Table S1. Titles and abstracts, followed by full texts, were dual-screened by 3 independent reviewers (Z.L., A.I., and A.R.) against predefined inclusion and exclusion criteria (Supplemental Table S2). Inclusion criteria for studies were as follows: publication in the period from 2012 to 2024; use of the WR; being written in English; having full-text availability; and being in the medical sciences field. Articles were excluded if they were theoretical or methodologic articles from statistical journals, or abstracts, conference papers, opinion pieces, editorials, or reviews. Discrepancies were resolved either by consensus, or if this could not be reached, by the opinion of the senior author (S.E.F.). Cohen's kappa was used to measure interrater reliability agreement between reviewers during the study selection process, including both title and/or abstract screening and full-text eligibility decisions. For title and abstract screening, values for Cohen’s kappa are 1.0 (Z.L. and A.I.), 0.75 (A.I. and A.R.), and 0.52 (Z.L. and A.R.). For full-text screening, values for Cohen’s kappa are 0.93 (Z.L. and A.I.) and 0.76 (Z.L. and A.R.).

### Data extraction

Bibliometric data were extracted by downloading metadata from PubMed and Web of Science and uploading it to the R package Bibliometrix (R Foundation, Vienna, Austria). Bibliometric data include title, journal, year of publication, citation count, and country. Each article was assigned a single country affiliation based on the affiliated country of the corresponding author.

Data were dually extracted by 2 independent reviewers (Z.L. and A.I.). Discrepancies were resolved by consensus. The following variables were collected: journal impact factor (retrieved from Web of Science Journal Citation Reports in August 2024); study design; funding; discipline; medical and/or surgical subspecialty; type of WR; use of win odds and/or win benefits; number of hierarchical outcomes used for WR; content of each outcome (mortality, CV mortality, hospitalizations, respiratory events and/or measures, stroke, renal events and/or measures, QoL measures, and complications); type of each outcome (binary, continuous, discrete, ordinal, recurrent, and multiple types); and number of outcomes. Time-to-event outcomes were defined as those in which both the occurrence and the timing of the event influenced the win determination (eg, time to death). Binary outcomes referred to those for which the presence or absence of an event by a specific time point determined the winner (eg, 30-day mortality, yes/no). If an article reported multiple WRs, the most prioritized WR was collected in data extraction (eg, if a study reported both an overall WR and a subgroup WR, the overall WR was used).

### Statistical analysis

Descriptive statistics were employed to summarize the data collected in this study. The normality of data was assessed using the Shapiro-Wilk test and a Q-Q plot. Variables that were approximately normally distributed were summarized using parametric methods (mean ± standard deviation), and non-normally distributed variables were summarized using nonparametric methods (median and interquartile range [IQR]—25th and 75th percentiles). The annual growth rate was calculated using the following compound formula: (ending value / starting value) ˆ (1 / n) – 1, where n is the number of years. Piecewise linear regression with the “segmented” package in R (R Foundation) was used to estimate changes in trends in the number of articles over time. Kendall’s correlational analyses were used to assess the associations between the year of publication, the journal impact factor, and the citation count. The cutoff values for weak, moderate, and strong Kendall’s tau correlation are 0.06, 0.26, and 0.49, respectively.^10^ All statistical analyses and graphical visualization using the package “ggplot2” were performed using R (R Foundation).

## Results

**Figure 1:**
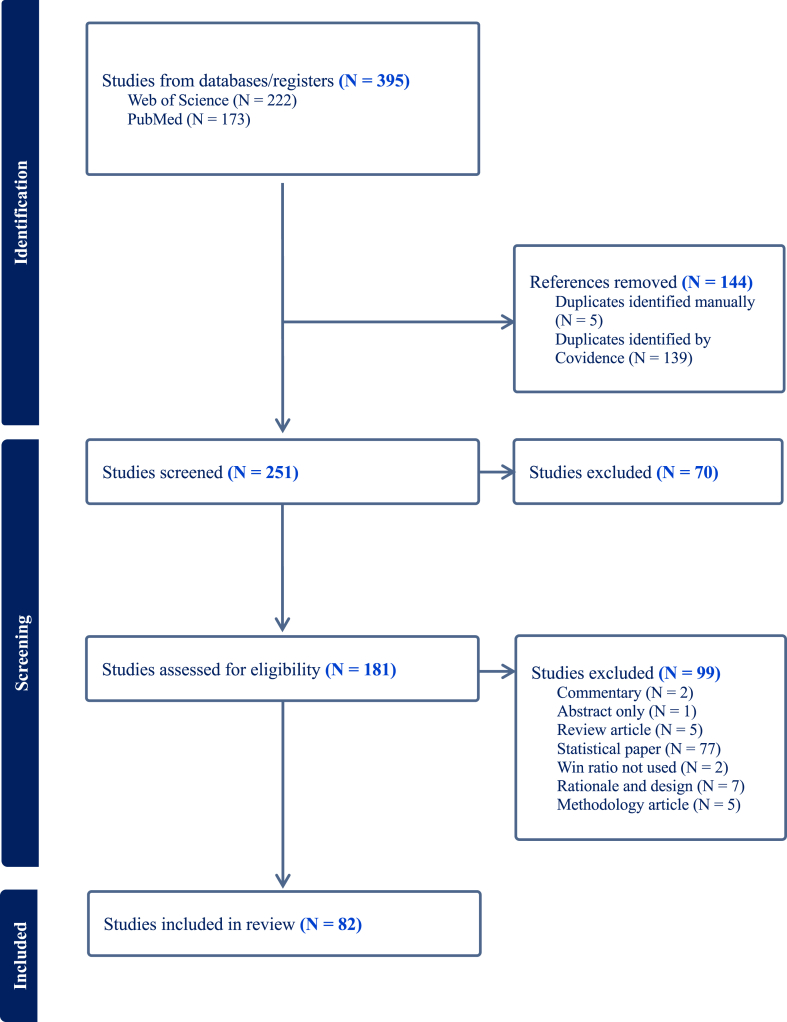
Flow diagram formatted in accordance with the Preferred Reporting Items for Systematic Reviews and Meta-Analyses (PRISMA) guidelines.

A total of 395 articles were retrieved from PubMed and Web of Science, and after de-duplication and screening, 82 articles were included in the final analysis (Fig. 1).

### Bibliometric information

As shown in Table 1, the articles were published in 48 different journals, with a median impact factor of 10.3 (25th percentile: 5.0; 75th percentile: 37.6) and an annual compounded growth rate of 30.2%. The publications involved a total of 1049 authors, with a median of 15 authors per article (IQR, 10, 26). The articles received a total of 4641 citations, with a median of 11 citations per article (IQR, 2.0, 40.8). Supplemental Figure S1 shows that the number of publications using the WR method has increased since 2020, with a difference in slope of 6.9 articles per year between the pre-2020 and post-2020 periods. The cumulative citations and number of articles per year are shown in Supplemental Figure S2. As shown in Supplemental Table S3, a moderate positive correlation (τ = 0.44, *P* < 0.001) was present between impact factor and citation count, and a strong positive correlation (τ = 0.53, *P* < 0.001) was present between citation count per year and year of publication.

**Table 1:** Bibliometric information of included articles

Variable	Value
Time range, y	2014 to 2024
Journals	48
Articles	82
Total authors	1049
Authors per article	15 (10, 26)
Total references	1647
References per article	25.5 (20.3, 32.0)
Impact factor	
Median	10.3 (5.0, 37.6)
Average	27.1 ± 32.2
Citations	
Median	11.0 (2.0, 40.8)
Average	61.1 ± 188.5
Total	4641
Annual growth rate,∗ %	30.2

Values are given as N, or average ± standard deviation, or median (25th percentile, 75th percentile), unless otherwise indicated.

∗ Annual growth rate was calculated using the following compound formula: (ending value / starting value) ˆ (1 / n) – 1, where the starting value (2014) is 2, the ending value (2024) is 28, and n (years) is 10.

As shown in Supplemental Table S4, the US was the leading country, based on the affiliated country of the corresponding author, in publishing articles using a WR, contributing 33 articles (40.2%), followed by Brazil (7 articles; 8.5%), the United Kingdom (6 articles; 7.3%), and France (6 articles; 7.3%). The private sector and industry were the primary funding sources for these studies, accounting for 51 occurrences (62.2%).

As shown in Table 2, 68 articles (82.9%) were in internal medicine; cardiology was the leading specialty employing the WR method, accounting for 46 articles (67.6%). RCTs were the most common study design, with 68 articles (82.9%). The top 5 relevant RCTs were as follows: Empagliflozin in Patients Hospitalized for Acute Heart Failure (EMPULSE), with 7 articles; A Study to Evaluate the Corvia Medical Inc IASD System II to Reduce Elevated Left Atrial Pressure in Patients with Heart Failure (REDUCE LAP-HF II), with 4 articles; Prospective Comparison of ARNI with ARB Given Following Stabilization in Decompensated HFpEF (PARAGLIDE-HF) with 3 articles; Dapagliflozin in Patients with Cardiometabolic Risk Factors Hospitalised with COVID-19 (DARE-19), with 3 articles; and Influenza Vaccination Strategy in Acute Coronary Syndromes (VIP-ACS), with 2 articles. Supplemental Table S5 shows the top 5 most-cited studies, which are as follows: Safety and Efficacy of Tafamidis in Patients with Transthyretin Cardiomyopathy (ATTR-ACT), with 1516 citations; EMPULSE, with 454 citations; Semaglutide in Patients with Heart Failure with Preserved Ejection Fraction and Obesity (STEP-HFpEF), with 347 citations; A Coronary Disease Trial Investigating Outcome With Nifedipine GITS (ACTION), with 345 citations, and Trial to Evaluate Cardiovascular Outcomes in Patients Treated with the Tricuspid Valve Repair System Pivotal (TRILUMINATE Pivotal), with 249 citations.

**Table 2:** Discipline and study design of articles

Variable	Value, N (%)
**Discipline**	
Internal medicine	68 (82.9)
Cardiology	46 (67.6)
Multiple subspecialties	12 (17.6)
Infectious diseases	3 (4.4)
Critical care	3 (4.4)
Nephrology	2 (2.9)
Respirology	1 (1.5)
Hematology	1 (1.5)
Surgery	10 (12.2)
General surgery	5 (50.0)
Cardiothoracic surgery	2 (20.0)
Urology	2 (20.0)
Transplantation surgery	1 (10.0)
Other	4 (4.9)
**Study design**	
Randomized controlled trial	68 (82.9)
Top 5 clinical trials∗	
EMPULSE	7 (10.3)
REDUCE LAP-HF II	4 (4.9)
PARAGLIDE-HF	3 (4.4)
DARE-19	3 (4.4)
VIP-ACS	2 (2.9)
Retrospective cohort	12 (14.6)
Prospective cohort	2 (2.4)

DARE-19, **Da**pagliflozin in Patients with Cardiometabolic **R**isk Factors Hospitalised with COVID-**19;** EMPULSE, Empagliflozin in Patients Hospitalized for Acute Heart Failure; PARAGLIDE-HF, **P**rospective Comparison of **AR**NI with **A**RB **G**iven Following Stabi**l**ization **i**n **De**compensated **HF**pEF; REDUCE LAP-HF II, A Study to Evaluate the Corvia Medical Inc IASD System II to **Reduce** Elevated **Left Atrial Pressure** in Patients with **Heart Failure**; VIP-ACS, Influenza **V**accination Strategy in **A**cute **C**oronary **S**yndromes.

∗ The top 5 trials with the highest number of publications. Secondary publications associated with these trials are included, resulting in multiple publications being counted for each trial.

### WR information

As shown in Table 3, the unmatched WR was the most prevalent type (57; 69.5%), followed by the matched (8; 9.8%) and stratified (6; 7.3%) approaches. Win odds and win benefits were used in 9 articles (11.0%) and 4 articles (4.9%), respectively. In RCTs, the WR was used typically for primary outcomes (48; 70.6%). A histogram of the magnitude of WR across studies is shown in Supplemental Figure S3. Most studies have a WR value close to 1.

**Table 3:** Win ratio information

Variable	Value, N (%)
**Type of win ratio**	
Unmatched	57 (69.5)
Matched	8 (9.8)
Stratified	6 (7.3)
Multiple types	11 (13.4)
**Use of win odds**	
Yes	9 (11.0)
No	73 (89.0)
**Use of win benefits**	
Yes	4 (4.9)
No	78 (95.1)
**Use of win ratio in RCTs**	
Primary outcome	48 (70.6)
Secondary outcome	9 (13.2)
Both primary and secondary outcomes	11 (16.2)

RCT, randomized controlled trial.

**Figure 2:**
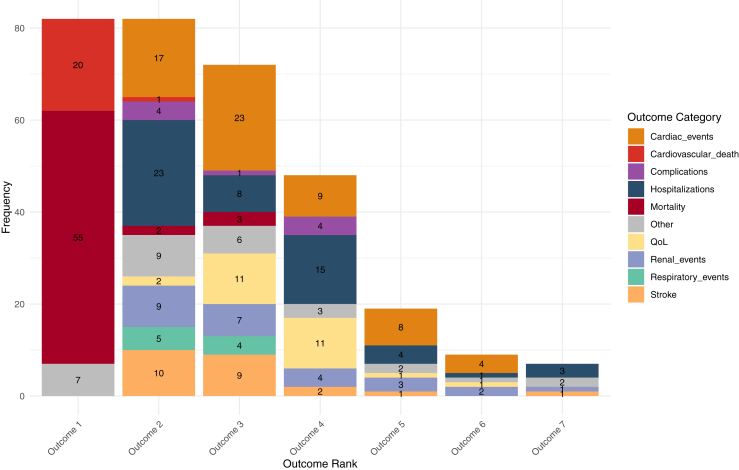
Distribution of hierarchical outcome categories. Each **bar** represents the total frequency of outcome categories for a specific outcome rank, including mortality, cardiovascular death, hospitalizations, cardiac events, stroke, renal events, respiratory events, quality of life (QoL), complications, and other outcomes.

As shown in Table 4, mortality (55; 67.1%) and CV death (20; 24.4%) were the most common highest-ranked outcomes in the composite hierarchy used for the WR method. The second-ranked outcomes in the hierarchy included hospitalizations (23; 28.0%) and cardiac events (17; 20.7%). In 3 studies, mortality was ranked third in the hierarchy. These studies prioritized short-term outcomes, such as 30- or 90-day mortality, and severe complications over long-term or overall survival. The distribution of outcomes from Table 4 is illustrated in Figure 2. As depicted in Supplemental Figure S4, time-to-event outcomes were the most commonly used type of variable in each individual rank (ie, outcome 1 to outcome 7) of composite hierarchies. In addition, as shown in Supplemental Figure S5, time-to-event outcomes have the highest frequency (N = 173; 54.9%) overall, followed by continuous (N = 50; 15.9%) and binary (N = 43; 13.7%) outcomes. These counts represent the total number of outcome variables across all hierarchical levels and all included studies, as many studies contributed multiple outcomes across different ranks and variable types.

**Table 4:** Hierarchical outcomes used for win ratio

Variable	Value, N (%)
**Outcome 1**∗**(N = 82)**	
Mortality	55 (67.1)
Cardiovascular death	20 (24.4)
Other	7 (8.5)
**Outcome 2 (N = 82)**	
Hospitalizations	23 (28.0)
Cardiac events and/or measures	17 (20.7)
Stroke	10 (12.2)
Renal events and/or measures	9 (11.0)
Respiratory events and/or measures	5 (6.1)
Complications	4 (4.9)
Mortality	2 (2.4)
Cardiovascular death	1 (1.2)
Quality of life	2 (2.4)
Other	9 (11.0)
**Outcome 3 (N = 72)**	
Cardiac events and/or measures	23 (31.9)
Quality-of-life measures	11 (15.3)
Stroke	9 (12.5)
Hospitalizations	8 (11.1)
Renal events and/or measures	7 (9.7)
Respiratory events and/or measures	4 (5.6)
Mortality	3 (4.2)
Complications	1 (1.4)
Other	6 (8.3)
**Outcome 4 (N = 48)**	
Hospitalizations	15 (31.3)
Quality-of-life measures	11 (22.9)
Cardiac events and/or measures	9 (18.8)
Complications	4 (8.3)
Renal events and/or measures	4 (8.3)
Stroke	2 (4.2)
Other	3 (6.3)
**Outcome 5 (N = 19)**	
Cardiac events and/or measures	8 (42.1)
Hospitalizations	4 (21.1)
Renal events and/or measures	3 (15.8)
Quality-of-life measure	1 (5.3)
Stroke	1 (5.3)
Other	2 (10.5)
**Outcome 6 (N = 9)**	
Cardiac events and/or measures	4 (44.4)
Renal events and/or measures	2 (22.2)
Hospitalizations	1 (11.1)
Quality-of-life measures	1 (11.1)
Other	1 (11.1)
**Outcome 7 (N = 7)**	
Hospitalizations	3 (42.8)
Renal events and/or measures	1 (14.3)
Stroke	1 (14.3)
Other	2 (28.6)

Definitions and examples for categories are as follows: - Mortality: all-cause mortality and mortality not specifically attributed to a cardiovascular cause (eg, death attributable to venous or arterial thrombosis); - Cardiovascular mortality: mortality with cardiovascular cause (eg, fatal coronary heart disease); - Hospitalizations: all-cause hospitalizations and length of stay; - Cardiac events and/or measures: cardiovascular events (eg, myocardial infarction), cardiac surgery, and change in cardiac markers (eg, N-terminal pro-B-type natriuretic peptide [NT-proBNP] levels); - Respiratory events and/or measures: respirology-related events (eg, pulmonary embolism) and need for respiratory support (eg, ventilation); - Stroke: stroke events and stroke-related symptom worsening (eg, worsening National Institutes of Health [NIH] Stroke Scale Score) - Renal events and/or measures: kidney-related treatment (eg, kidney replacement therapy), renal markers (eg, changes in creatinine levels), kidney function (eg, significant decreases in estimated glomerular filtration rate); - Quality-of-life measures: patient-reported outcomes (eg, Kansas City Cardiomyopathy Questionnaire Overall Summary Score) and functional assessments (eg, New York Heart Association functional classification); and - Complications: surgical complications (eg, bleeding events).

∗ Outcomes are numbered according to their rank in the outcome hierarchy.

The highest-ranked outcomes were more commonly mortality-related outcomes, whereas nonfatal outcomes and QoL categories were more commonly lower-ranked outcomes (Supplemental Table S6). The WR was used as the primary analysis in 43 studies (52.4%) and was used as a *post hoc* analysis or secondary analysis in 39 studies (47.6%). In addition, 59 studies (72.0%) were unique, whereas 23 studies (28.0%) had at least one other publication derived from the same study.

## Discussion

This bibliometric review highlights a marked increase in the use of the WR method, especially unmatched WR, in clinical research since its introduction in 2012, with a notable rise since 2020. Most studies using the WR were RCTs in cardiology. Mortality was the single, most common highest-ranked outcome, and time-to-event variables were the most frequently used type across all ranks in composite hierarchies.

**Figure 3:**
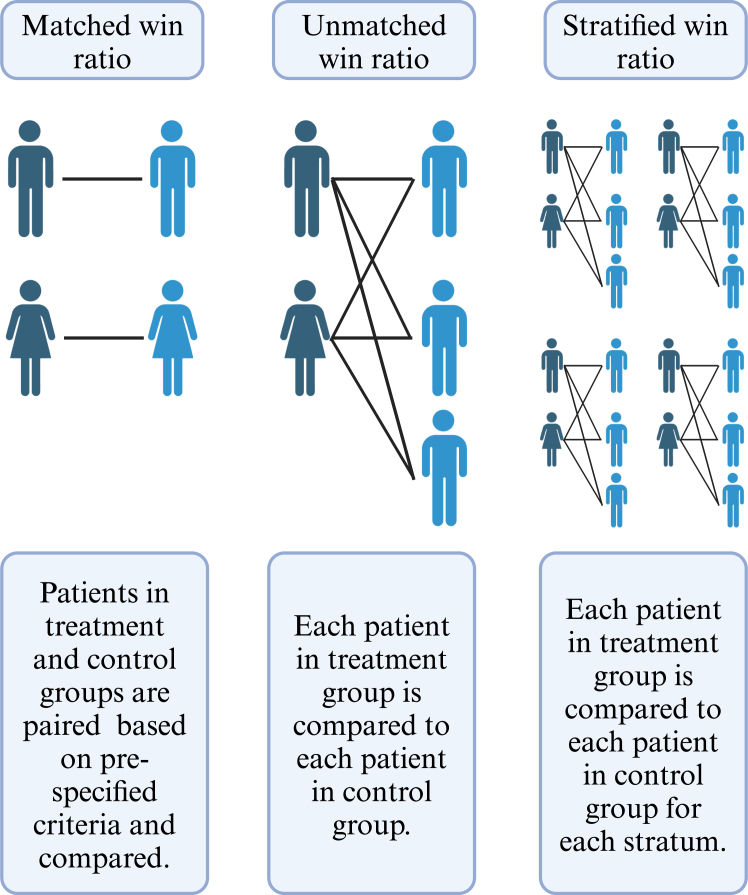
Types of pairwise comparison used to calculate win ratio (WR). The figure illustrates 3 approaches for comparing patients in treatment and control groups using the WR method. In the matched WR approach, patients in the treatment and control groups are paired based on prespecified matching criteria, such as age, sex, or baseline characteristics, and compared within these pairs. In the unmatched WR approach, each patient in the treatment group is compared with every patient in the control group, generating all possible pairwise comparisons. The stratified WR approach divides patients into strata based on specific characteristics, such as disease severity. Within each stratum, every patient in the treatment group is compared with every patient in the control group, and results are aggregated across strata. **Arrows** represent comparisons between patients in the treatment group and the control group.

In our study, the unmatched WR is the most represented across studies. The 3 main types of WR^2^ are as follows: (i) the matched WR, which involves pairing patients based on important prespecified variables, allowing for direct comparisons within these matched groups; (ii) the unmatched WR, for which each patient in the treatment group is compared to every patient in the control group; and (iii) the stratified WR, which divides patients into strata, with a WR calculated for each stratum (Fig. 3). These stratum-specific WRs are then aggregated to provide an overall result that accounts for different subgroups within the study population. The different WR approaches offer distinct advantages depending on the study context. The unmatched WR is simple to implement and commonly is used when randomization ensures baseline balance.^2^ However, this approach may introduce bias in nonrandomized studies or trials with baseline imbalances.^2^ In such cases, matched or stratified WRs may be more appropriate. The **matched approach** addresses this bias by pairing individuals based on similar baseline characteristics, reducing confounding.^2^ However, this approach rarely is used in practice, due to the difficulty of prospectively defining all relevant matching variables.^2^ The stratified WR allows for subgroup-specific comparisons while preserving randomization.^2^ The selection of approach should be driven by study design, baseline risk considerations, and the intended estimand.

The rise in the number of articles after 2020 is likely due to several factors. One potentially important driver was COVID-19-related studies. For instance, the Rivaroxaban to Prevent Major Clinical Outcomes in Non-Hospitalised Patients with COVID-19 (CARE-COALITION VIII) trial evaluated rivaroxaban 10 mg daily for 14 days vs routine care in 660 nonhospitalized COVID-19 patients, using both a composite primary endpoint and WR analysis that included COVID-19 hospitalization.^11^ Moreover, a notable challenge during the COVID-19 pandemic was disruptions in research and incomplete data collection.^12^ The WR method may have allowed comparisons to proceed even if data for lower-priority events were missing, as long as higher-ranked data, such as mortality, were available.

Similarly, the prevalence of the WR method in cardiology trials also can be attributed to several factors. CV disease is a leading cause of death worldwide, and CV trials account for a substantial proportion of clinical research activity, making up approximately 10% of all trial participants, according to data from ClinicalTrials.gov.^13^ In addition, many CV trials use composite outcomes, such as major cardiac or cerebral adverse event (MACCE), usually defined as the time to all-cause or CV death, nonfatal myocardial infarction, or nonfatal stroke, making them well suited for WR analysis that requires a hierarchy of composite outcomes.^14^ In addition, early adoption and the successful application of the WR, particularly following the landmark 2012 paper by Pocock et al.,^1^ likely have encouraged its continued use in this field.

However, the utility of the WR is not limited to CV trials. Our study shows that it also has been used in observational studies, although its application in these settings introduces specific challenges. Observational studies have numerous confounding factors that are more difficult to control than those in randomized trials.^15^ As a result, the unmatched WR approach, in which every patient is compared to one another in 2 groups, lacks control of these confounders, which can skew the results.^15^ To mitigate this issue, matching patients in pairs based on preexisting factors, using methods such as propensity-score matching, can provide a more balanced comparison between treated and untreated individuals for WR analysis.^15^ For instance, in an observational study by Gorji et al. comparing multi-visceral resection with tumour-only resection in cases of liposarcoma, a matched WR approach was used, controlling for covariates such as tumour involvement, grade, stage, age group, and comorbidities.^16^

In terms of the receptivity of government regulators, a meta-analysis of 18 phase III pulmonary arterial hypertension trials submitted to the US Food and Drug Administration from 2000 to 2015 incorporated win statistics,^17^ suggesting that the agency has reviewed data using the WR, although the agency’s position on use of the WR as a primary estimand remains unclear. The European Medicines Agency has not endorsed the WR explicitly but supports advanced statistical methodologies consistent with International Council for Harmonisation guidance on statistical principles in clinical trials.^18^ Health Canada has not issued specific guidance on the WR, but the organization generally aligns with internationally harmonized standards that support innovative approaches.^19^

In terms of outcome types in the WR hierarchies, the distribution shows that a higher proportion of objective clinical endpoints (eg, mortality and hospitalization) are at the top levels, with a shift toward patient-reported and QoL measures in lower-ranked outcomes. This arrangement suggests that clinical trials employing the WR still prioritize traditional endpoints over patient-centred ones, even though the methodology offers the flexibility to incorporate both. Although objective clinical measures remain central in clinical trials, patients often place more emphasis on QoL measures.^7^ In our study, QoL measures were found to include both objective and subjective assessments, including the Kansas City Cardiomyopathy Questionnaire (KCCQ),^20^ the 6-minute walking distance test,^21^ the Minnesota Living with Heart Failure Questionnaire (MLHFQ),^22^ New York Heart Association (NYHA) functional class,^23^ and a discharge status that includes the ability to resume normal activities.^24^ Appreciation is growing for use of patient-centred outcomes in research, with the WR being applied to analyze these composite endpoints. For instance, the **Re**vacularization **Ch**oices **A**mong Under-**R**epresented **G**roups **E**valuation (RECHARGE: Minorities) Trial (NCT06399705) is a multicentre, randomized study comparing coronary artery bypass grafting (CABG) and percutaneous coronary intervention (PCI) in minority populations with coronary artery disease.^25^ The trial focuses on patient-reported outcomes such as QoL, using tools such as the Short-Form 12-Item Survey-version 2 (SF-12v2), the Seattle Angina Questionnaire, and Patient-Reported Outcomes Measurement Information System (PROMIS)-29.^25^ The shift toward integrating patient-centred outcomes highlights the growing importance of aligning clinical research with the experiences of patients. Future WR analyses could benefit from engaging patients and stakeholders to prioritize outcomes that align with patient values, such as emphasizing QoL improvements, vs making changes in surrogate measures.

The process of defining hierarchies for composite endpoints in WR analyses requires careful consideration of clinical relevance and stakeholder input. Expert consensus often is employed to align with clinical guidelines and ensure that the hierarchy reflects established medical priorities. At the same time, incorporating patient perspectives helps ensure that outcomes of personal significance are represented,^7^ although variability can complicate standardization.^2^ Debate is ongoing regarding which components should be included in hierarchical composite outcomes. Sensitivity analyses can help address this concern. For example, in the **Dapa**gliflozin **H**eart **F**ailure (DAPA-HF) and Dapagliflozin Effect on Symptoms and Biomarkers in Diabetes Patients With Heart Failure (DELIVER) trials evaluating kidney-related outcomes, all-cause mortality was placed at the top of the hierarchy.^26^ Recognizing that all-cause mortality may not be kidney-specific, investigators conducted sensitivity analyses excluding mortality to assess the robustness of their findings.^26^ A combined approach that integrates expert opinion, patient input, and other stakeholder perspectives, along with sensitivity analyses to evaluate robustness, is likely to be an ideal strategy for developing outcome hierarchies. Although the WR is used increasingly, our study found that in over 45% of studies, the WR served as a secondary or *post hoc* analysis rather than the primary analysis. This usage likely reflects the method’s novelty and the dominance of traditional approaches in trial design. Planning a clinical trial with the WR as the primary endpoint requires several methodologic decisions, such as how to rank outcomes, how to handle tied comparisons, and how to determine statistical power. These considerations differ from those in traditional time-to-event analyses. As use of the WR grows, consensus on design standards is still emerging.

The most used variables in WR analyses were time-to-event outcomes, followed by binary and continuous outcomes. Typically, time-to-event outcomes determine the winner by taking into account which patient experiences the event later. For example, in an RCT assessing the efficacy of an integrated care approach in improving the prognosis of AF patients by Romiti et al., the winner was the patient who either did not experience the event or did experience it later.^27^ However, some studies treat events such as mortality as a binary variable, with the presence or absence of the event determining the winner, as seen in a retrospective cohort study comparing minimally invasive and open pancreaticoduodenectomy by Beal et al., in which the winner for each of the first 2 ranks in the WR hierarchy was determined as “no mortality at 30 days” followed by “no mortality at 3 years.”^28^ Continuous outcomes also are included frequently in WR hierarchies, but they may be classified as binary if a threshold is applied. For example, in the study by Kandzari et al., one of the hierarchical outcomes was the change in 24-hour mean ambulatory systolic blood pressure from baseline, and a 5-mm Hg threshold was used to determine winners between groups.^29^ Together, these approaches demonstrate the adaptability of WR for handling different variable types.

Although the WR provides a flexible approach for analyzing various outcome types, it is not without limitations. Ajufo et al. and Grubic & Ezekowitz have discussed several limitations of the WR and suggested potential solutions.^2^^,^^30^ First, the hazard ratio (HR) denotes the ratio of the event rate between the treatment and control groups, but the WR cannot be interpreted in the same way in terms of overall treatment effect over time.^2^ Although in a review of 16 CV trials using WR, Ferreira et al. found that 1/WR and HR yielded similar numeric values in RCTs, such as in the , **P**rospective Comparison of **AR**Ni With **A**CEi to **D**etermine **I**mpact on **G**lobal **M**ortality and Morbidity in **H**eart **F**ailure (PARADIGM-HF) trial comparing sacubitril and/or valsartan to enalapril^6^; this similarity does not imply equivalence in meaning or interpretation.

In addition, although outcomes are ordered by clinical priority in the hierarchy, the WR treats each component equally in the final analysis. As a result, statistically significant findings may be driven primarily by wins on outcomes that are less clinically meaningful.^30^ This limitation becomes especially relevant when subjective or short-term endpoints, such as certain QoL measures, are included in the hierarchy. For example, early wins on symptom improvement or functional status could obscure longer-term benefits or harms that emerge from more-definitive outcomes such as mortality or major adverse events. This risk is amplified in studies with limited follow-up duration, in which hard outcomes may not have had sufficient time to accrue. Moreover, wins in time-to-event outcomes based on time difference may not be clinically significant if the time difference is small (eg, 1 day).^2^^,^^30^ Reporting estimates of time-dependent outcomes, as in traditional survival analysis,^2^ or setting a threshold for clinically meaningful differences may help better evaluate clinical significance. Moreover, the WR does not account for tied outcomes.^2^ In standard WR analyses, tied pairs (ie, neither patient has a clearly better outcome) are excluded from both the numerator and denominator, as the WR is produced by dividing the number of wins in the treatment group by the number of wins in the control group.^2^ To address the limitations, alternative approaches such as use of win odds (WO) and win benefit (WB) have been proposed. The WO approach addresses the issue of ties by adding one-half the number of all ties to the number of wins for each arm,^2^; the WB represents the percentage of pairwise comparisons in which one group demonstrates a better outcome than does the other.^31^ Despite their potential advantage, WO and WB were found to be used in only a small fraction of articles in this study, likely reflecting their recent (in 2023) introduction.^31^ As these methods gain recognition, their use may become more common in clinical research.

The statistical evaluation of the WR relies on established methods, but the lack of standardized effect size measures presents challenges for its interpretation in clinical research. The significance of WR often is assessed by statistical tests, such as the generalized Wilcoxon rank-sum test, the *Z*-test, or *U* statistics.^31^ According to Pocock et al., a WR > 1 indicates a beneficial effect of the treatment group,^1^ but no numeric thresholds (eg, 1.5 or 2.0) for strong, moderate, or weak effects have been defined. For example, the DAPA-HF and DELIVER trials, which evaluated the efficacy of dapagliflozin (a sodium glucose transporter 2 [SGLT2] inhibitor) in patients with heart failure, reported a combined WR of 1.10 (95% confidence interval: 1.06-1.15) with a *P* value < 0.001.^26^ Although the *P* value indicates statistical significance, the value of WR may suggest that this significance has limited clinical relevance. Future research should focus on standardizing effect size measures and establishing benchmarks for interpreting the WR. The WR is particularly well-suited for trials with composite outcomes or clearly defined hierarchies of outcome importance (eg, prioritizing death over hospitalization). Conversely, for simpler endpoints, such as a single binary or time-to-event outcome, traditional measures such as the HR or the odds ratio that have established statistical and interpretive benchmarks may be more straightforward and effective. Scientific stakeholders should consider WR evidence alongside traditional methods, appreciating the WR’s unique value while recognizing its limitations. The interpretation of the WR should reflect the broader context of clinical significance, with careful attention to effect size, confidence intervals, and statistical significance.

### Limitations

Given the rapidly evolving nature of the WR field, additional papers likely have been published since the initial search was conducted. In addition, the classification of event-type variables (eg, mortality) as either time-to-event or binary is unclear in some studies. These variables were assumed to be time-to-event, as this classification is the most probable, but such assumptions may introduce bias. Additionally, some articles report multiple WRs in the same study; however, for the purpose of descriptive statistics, the study’s highest-priority outcome WR was used whenever applicable.

### Conclusions

The WR provides a valuable framework for analyzing hierarchical outcomes in biomedical research, in which composite outcomes are frequently used, particularly in CV trials. However, broader adoption of the WR requires addressing its key limitations, which include the handling of ties and the standardization of effect-size interpretation. Enhancing the WR's robustness could involve integrating complementary metrics such as the WO and WB, as well as involving patients and stakeholders in defining and prioritizing outcomes. These improvements would strengthen the WR’s methodologic rigour, clinical relevance, and alignment with patient-centred care in CV and other medical fields.
