# Supplementary material for: Win Ratio in Biomedical Science: A Bibliometric Analysis
Source: CJC Open. 2025 May 21;7(8):1097–107. doi: 10.1016/j.cjco.2025.05.006 (PMC12399148; doi:10.1016/j.cjco.2025.05.006)
Supplement: Supplementary Material [file mmc1.pdf]

Supplemental Table S1. Search strategy for Web of Science and PubMed

Database	Search string
Web of Science	TS = ("win ratio" OR "win statistic*" OR “win odds” OR “Finkelstein Schoenfeld”)
PubMed	"win ratio" OR "win statistic*" OR “win odds” OR “Finkelstein Schoenfeld”

Time range was restricted to 2012-2024

Supplemental Table S2. Inclusion and exclusion criteria

Inclusion criteria	Exclusion criteria
- Study is published in 2012-2024<br>- Win ratio is used<br>- Article is written in English<br>- Full text is available<br>- Study is in the field of medical sciences	- Theoretical and methodological articles from statistical journals[1]<br>- Studies that do not fulfill the inclusion criteria (e.g. abstracts, conference papers, opinion papers and editorial papers)<br>- Review articles

[1]Given the biomedical focus of this study, articles from statistical journals that primarily explored methodological aspects of the win ratio were excluded.

Supplemental Table S3. Kendall correlational analyses between numerical variables

	Citation count	Impact factor	Year of publication
Citation count		**0.44**	**-0.46**
Impact factor	**0.44**		-0.02
Year of publication	**0.53**[1]	-0.02	

Bolded values indicate statistically significant correlations with P < 0.001

[1]Citation counts were adjusted for time since publication by dividing total citations by the number of years since publication (i.e., 2024 − publication year) to calculate citation rate per year.

Supplemental Table S4. Characteristics of authors and funding sources

Variable	Value
**Top 5 countries[1]**	**N (%)**
United States	33 (40.2)
Brazil	7 (8.5)
United Kingdom	6 (7.3)
France	6 (7.3)
Canada	3 (3.7)
**Funding[2]**	**N**
Private sector and industry	51
Government agencies	22
Academic institutions and hospitals	7
No funding	5
Not specified	5
Peer-reviewed foundations	3
Philanthropic foundations and non-profit organizations	2

[1]Each article is assigned a single country affiliation based on the affiliated country of the corresponding author.

[2]Each study may have multiple sources of funding; therefore, the total counts and percentages may exceed 82 and 100%, respectively.

Supplemental Table S5. Characteristics of top 5 cited articles

Author, year of publication	Study acronym	Title	Study design	Discipline	Sample size	Number of citations	Journal	Outcome hierarchy evaluated by win ratio
Maurer et al, 2018	ATTR-ACT	Tafamidis Treatment for Patients with Transthyretin Amyloid Cardiomyopathy	Randomized controlled trial	Internal medicine, Cardiology	441	1516	NEJM	All-cause mortality, frequency of cardiovascular-related hospitalization
Voors et al., 2019	EMPULSE	The SGLT2 inhibitor empagliflozin in patients hospitalized for acute heart failure: a multinational randomized trial	Randomized controlled trial	Internal medicine, Cardiology	530	454	Nature Medicine	All-cause mortality, number of heart failure events and time to first heart failure event and a 5 point or greater difference in change from baseline in the KCCQ-TSS
Kosiborod et al., 2023	STEP-HFpEF	Semaglutide in Patients with Heart Failure with Preserved Ejection Fraction and Obesity	Randomized controlled trial	Internal medicine, Cardiology	516	347	NEJM	All-cause mortality, heart failure events, difference in change in the KCCQ-CSS and 6-minute walk distance
Lopes et al., 2021	ACTION	Therapeutic versus prophylactic anticoagulation for patients admitted to hospital with COVID-19 and elevated	Randomized controlled trial	Internal medicine, Infectious disease	615	345	LANCET	Time to death, duration of hospitalisation and duration of supplemental oxygen

		D-dimer concentration (ACTION): an open-label, multicentre, randomised, controlled trial						
Sorajja et al., 2023	TRILUMINATE Pivotal	Transcatheter Repair for Patients with Tricuspid Regurgitation	Randomized controlled trial	Internal medicine, Cardiology	350	249	NEJM	All-cause mortality or tricuspid-valve surgery, hospitalization for heart failure and an improvement in quality of life as measured with the KCCQ at the 1-year follow-up

KCCQ-TSS: Kansas City Cardiomyopathy Questionnaire Total Symptom Score; NEJM: New England Journal of Medicine; KCCQ-CSS: Kansas City Cardiomyopathy Questionnaire Clinical Summary Score

Supplemental Table S6. Contingency table of outcomes by mortality and quality of life measures

	**Mortality**	**Non-mortality**
**Outcome 1**	75	7
**Outcome 2**	3	79
**Outcome 3**	3	69
**Outcome 4**	0	48
**Outcome 5**	0	19
**Outcome 6**	0	9
**Outcome 7**	0	7
	**Quality of life measures**	**Non-quality of life measures**
**Outcome 1**	0	82
**Outcome 2**	2	80
**Outcome 3**	11	61
**Outcome 4**	11	37
**Outcome 5**	1	18
**Outcome 6**	1	8
**Outcome 7**	0	7

Supplemental Figure S1. Piecewise linear regression of the number of articles over time. The black points represent the observed data, the blue lines indicate the fitted regression lines before and after the breakpoint in 2020 (red dashed line). The x-axis indicates the year, and the y-axis represents the number of articles published.

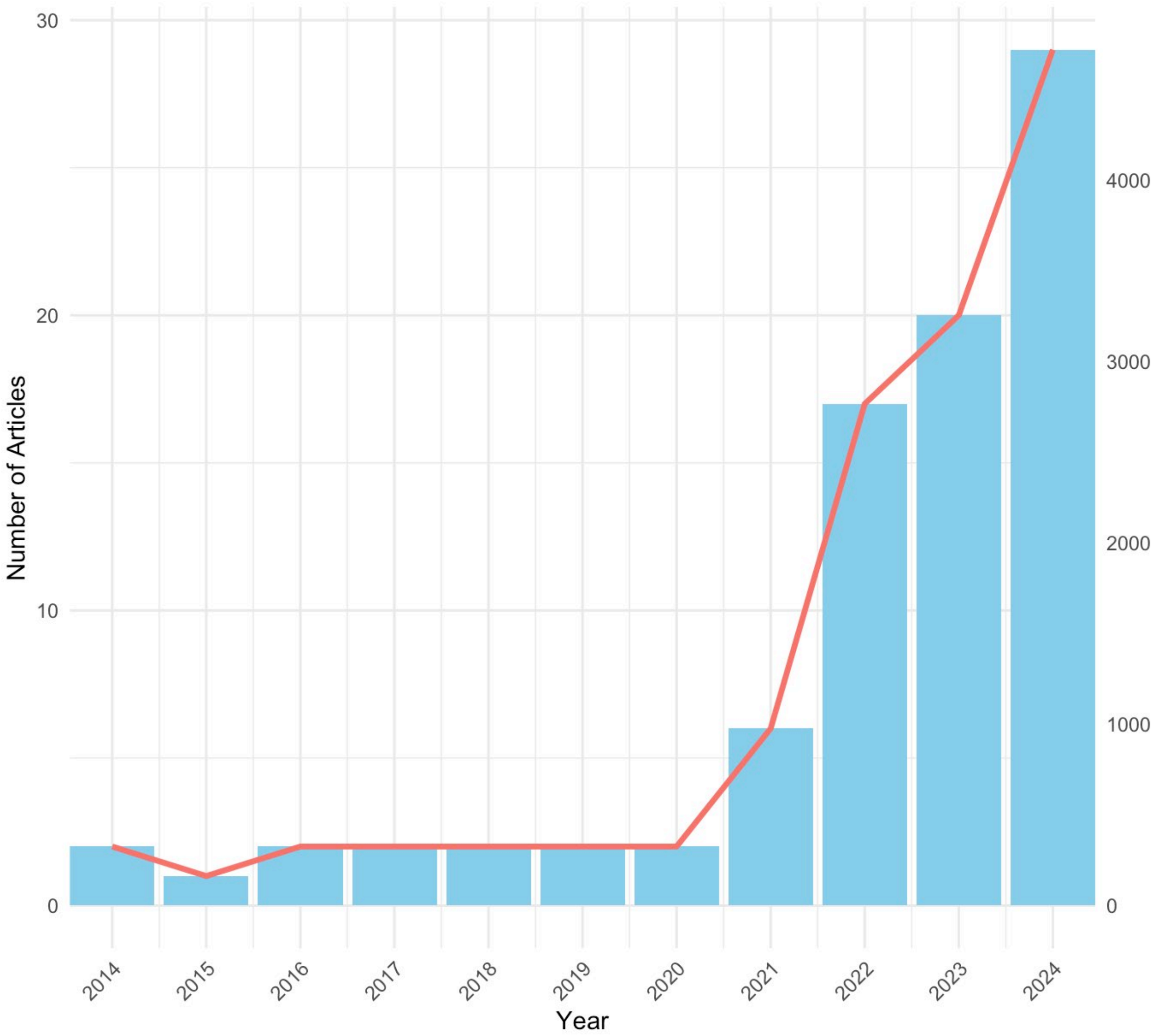

Supplemental Figure S2. Cumulative citations and number of articles per year. The x-axis represents the year, and the y-axis represents the number of cumulative citations and number of articles.

Supplemental Figure S3: Histogram of win ratio magnitudes across studies. The x-axis represents the win ratio magnitude with intervals (bin width) of 0.05, while the y-axis indicates the frequency of studies in each win ratio interval.

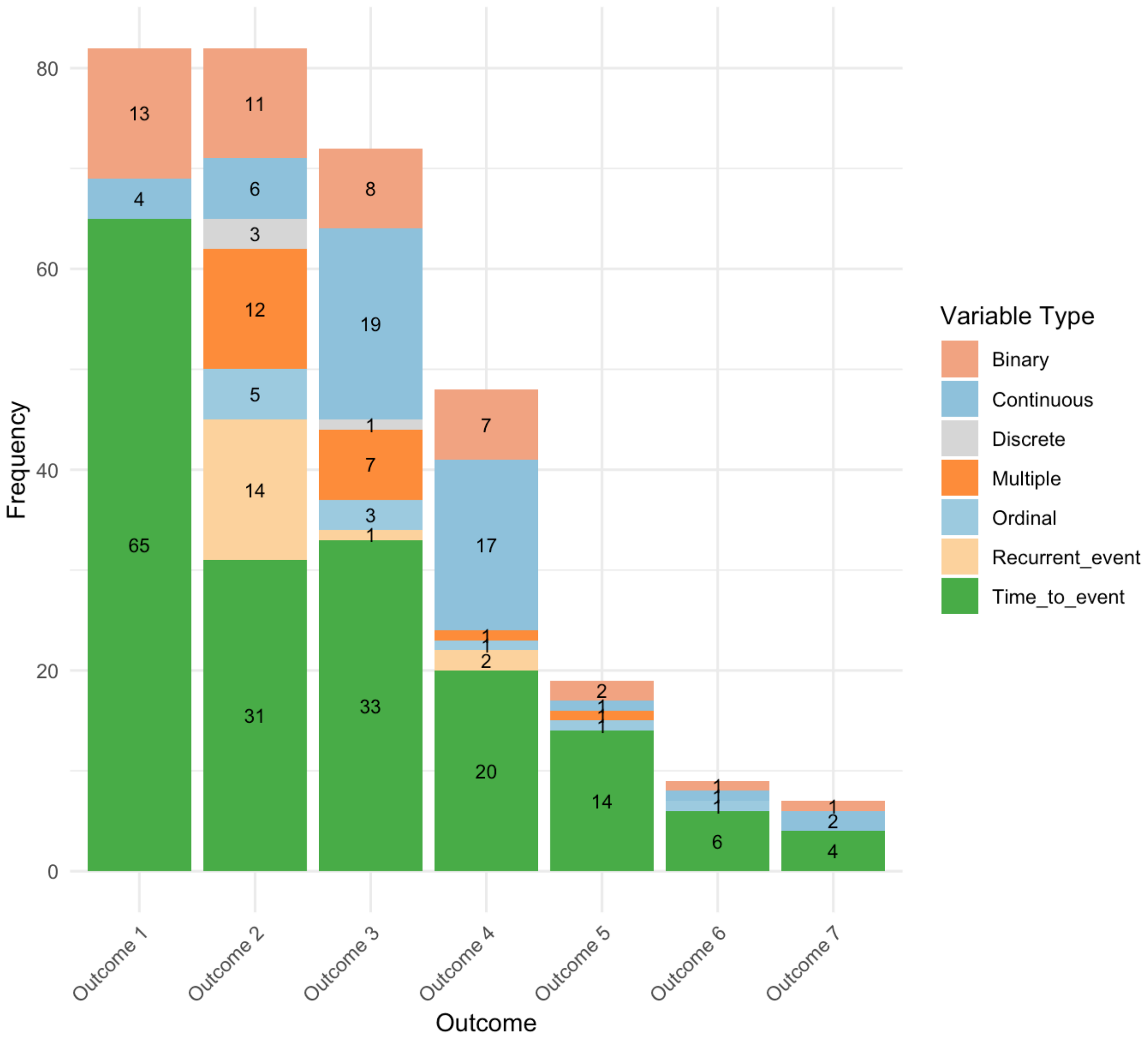

Supplemental Figure S4. Distribution of the type of outcomes in the hierarchy. Each bar represents the total frequency of variable types for a specific outcome. The variable types are labelled within the chart with the corresponding frequency values. Quality of life outcomes are classified into different types depending on the context. If a threshold is involved (e.g. improvement of ≥ 15 points in KCCQ score), the outcome is classified as binary. If the outcome represents a change in score or numerical value (e.g., differences in 6-minute walk distance), it is classified as continuous. If the variable falls into a category or class (e.g., NYHA functional class), it is classified as ordinal. A variable is assigned as discrete if it is a numerical variable that is not continuous (e.g. “number of complications”). A variable is classified as "multiple" if it falls into more than one category. For example, if the winner for hospitalization is defined as the person who was hospitalized later and/or had fewer hospitalizations, then hospitalization is considered both a time-to-event and a recurrent event variable. In this case, it is assigned as multiple.

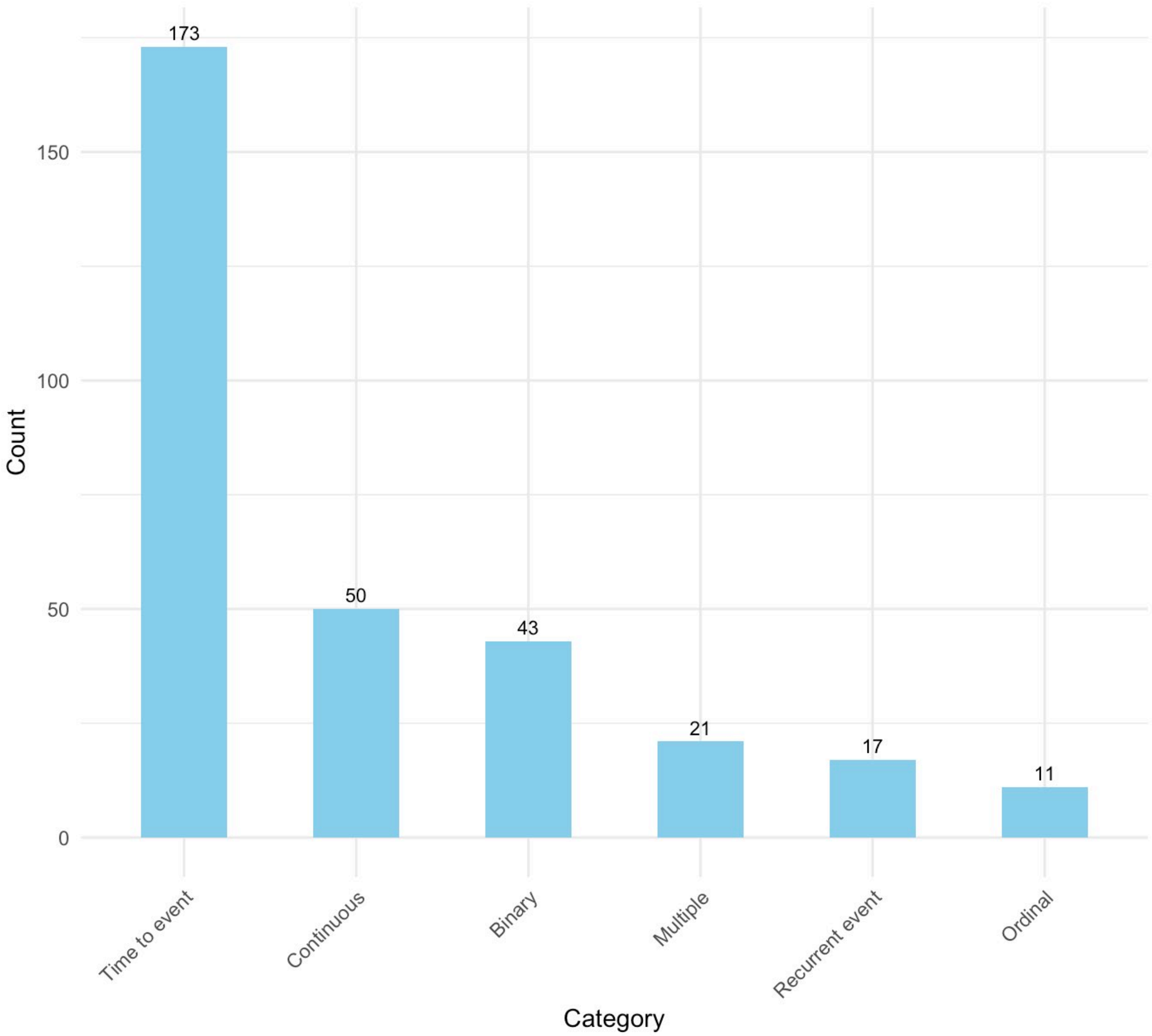

Supplemental Figure S5. Frequency of types of outcomes in the hierarchy. The x-axis represents the categories, while the y-axis represents the count for each category. Each bar is labelled with its respective count.
